# Refractive factors affecting the persistence of anisometropia in preschool-aged children

**DOI:** 10.1007/s00417-025-06891-w

**Published:** 2025-06-27

**Authors:** Ran Moshkovsky, Itay Nitzan, Michael Kinori, Oriel Spierer

**Affiliations:** 1 Department of Ophthalmology, Wolfson Medical Center, Holon, Israel; 2 Gray Faculty of Medical and Health Sciences, Tel Aviv University, Tel Aviv, Israel; 3 Department of Ophthalmology, Hadassah Medical Organization, Jerusalem, Israel; 4 Faculty of Medicine, Hebrew University of Jerusalem, Jerusalem, Israel; 5 Department of Ophthalmology, The Goldschleger Eye Institute, Sheba Medical Center, Ramat Gan, Israel

**Keywords:** Anisometropia, Astigmatism, Hyperopia, Myopia, Refractive error

## Abstract

**Purpose:**

Pediatric vision screening programs can detect children with amblyopic anisometropia. We aim to determine which refractive factors may be associated with the persistence of anisometropia in this population.

**Methods:**

A cohort analysis included all children aged one to six years, evaluated for refractive status between 2012 and 2022 at the Maccabi Healthcare Services. Refractive data were collected at presentation and at a subsequent follow-up visit. Logistic regression models were used to analyze relationships between baseline refractive error and anisometropia at follow-up.

**Results:**

Of 35,854 children evaluated, 6.6% (*n* = 2,358) had anisometropia at presentation. Those with a baseline anisometropia of ≥ 3.0 diopters exhibited a higher prevalence of anisometropia at the last follow-up compared to those with a baseline anisometropia of a lesser magnitude; when the more ametropic eye was hyperopic (73.5% vs. 41.4%, respectively, *p* < 0.001) or myopic (62.5% vs. 41.8%, respectively, *p* = 0.009). Adjusted odds ratios for anisometropia at follow-up increased with greater levels of baseline anisometropia with the more ametropic eye being hyperopic (OR = 3.75, 95% CI 2.57 – 5.48) or myopic (OR = 2.19, 95% CI 1.11 – 4.33). A higher degree of hyperopia, myopia and astigmatism at baseline demonstrated similar patterns.

**Conclusion:**

In anisometropic preschool-aged children, higher degrees of baseline anisometropia, hyperopia, myopia and astigmatism, are associated with a higher prevalence of future anisometropia. The likelihood for later anisometropia increases with greater levels of baseline anisometropia, hyperopia, myopia and astigmatism.

**Key messages:**

***What is known***

- Higher refractive errors, lifestyle factors and educational levels have been considered as risk factors for anisometropia. Severe anisometropia at young age is more prone to persist.

***What is new***

- In this cohort of 2,358 anisometropic preschool-aged children, the likelihood for anisometropia at the last follow-up has increased considerably when, at baseline, the more ametropic eye was hyperopic.
- It has also increased with astigmatic hyperopia, which possibly underscores the combined effect of astigmatism and hyperopia on anisometropia persistence.
- These findings were independent of socio-economic status, body mass index, and country of birth.

**Supplementary Information:**

The online version contains supplementary material available at 10.1007/s00417-025-06891-w.

## Introduction

The asymmetry in spherocylindrical refractive error between both eyes is termed anisometropia and has been observed to be more commonly found in cases of high ametropia [1]. Early detection and intervention are crucial for managing anisometropia's impact on early visual development [2]. Most cases of infantile anisometropia are axial in nature [3]. Although there is no universally accepted dioptric value for clinically significant anisometropia, most researchers define it as an interocular spherical equivalent (SE) difference of 1.0 diopter (D) or greater. Based on this threshold, the prevalence of anisometropia varies widely upon age, gender, and ethnicity and may range from 1.3 to 11.2 percent across different studies [4]. Comparing between prevalence studies is challenging due to diverse demographics and dissimilar methodologies.

Higher refractive errors, lifestyle factors and educational levels have been considered as risk factors for anisometropia [5–7]. Longitudinal studies demonstrated increased prevalence of anisometropia after children begin to attend school [8]. It has been postulated that extended near-work period and higher frequency of myopia with increasing age are related to each other and may explain the variation of anisometropia during school age [9, 10]. Other studies have shown that most of anisometropia progression occurs in the first few years of life before becoming stable. Some cases spontaneously resolve as the eyes naturally become emmetropic, while severe anisometropias at young age are more prone to persist [11, 12]. This can suggest that although older children may present with a large anisometropic refractive error, it was probably present at preschool-age.

The current study was conducted on a large sample of anisometropic young children covering a broad range of refractive errors to determine which refractive factors may be associated with the persistence of anisometropia in this population. This information could later be used in identifying who is most likely to benefit from intensive follow-up regimen, early refractive correction, or alternative prophylactic therapy.

## Methods

### Study design and population

A population-based retrospective cohort analysis was performed including all children aged one to six years with a minimum two-year follow-up between 2012 and 2022 at the Maccabi Healthcare Services (MHS). In Israel, all citizens are covered by a national health law which mandates enrollment in one of four official Health Maintenance Organizations (HMOs) and by so, allows subsidized access to medical treatment. Serving 26.7% of the national population, MHS is Israel’s second-largest HMO. It holds a centralized electronic database comprising longitudinal records for over 2.5 million individuals, 32% of whom are children (0–16 years). This database elaborates socio-demographic characteristics, socio-economic status (SES), country of birth, outpatient international classification of diseases (ICD)−10 diagnoses and clinical evaluations. These facts make our cohort representative of a large fraction of the pediatric patients who underwent at least two ophthalmologic examination involving refractive measurements. Children with chromosomal anomalies, ocular trauma, keratoconus, and cataract, (defined by ICD-10 codes) were excluded. The remaining children, aged one to six years with anisometropia at presentation, were eligible for refractive data analysis. As all eligible patients in the database were included, no sample size calculation was performed. The minimum anisometropia value considered as an inclusion criterion was defined by an interocular SE difference of at least 1.0 D. SE, calculated by adding the sum of the sphere power with half of the astigmatic power measured in D, was used to quantify the refractive error. To allow for potential changes in refractive error to manifest over time, a minimum two-year follow-up visit for subsequent refractive evaluation was set. This study was approved by the Institutional Review Board (MHS IRB committee, protocol number MHS-0073–21) and was conducted according to the Declaration of Helsinki principles.

### Study variables

Categorization into type and degree of refractive error was based on the eye exhibiting the higher degree of ametropia. Anisometropia was defined by an interocular SE difference of at least 1.0 D and was further categorized into moderate (1.0 D ≤ SE < 3.0 D) and severe (SE ≥ 3.0 D) [13]. Hyperopia was defined as a SE of 1.0 D or greater and was further categorized into mild (1.0 D ≤ SE < 3.0 D), moderate (3.0 D ≤ SE < 5.0 D) and severe (SE ≥ 5.0 D) [14]. Myopia was defined as a SE of −0.5 D or lesser, and was further categorized into mild (−3.0 D < SE ≤ −0.5 D), moderate (−6.0 D < SE ≤ −3.0 D) and severe (SE ≤ −6.0 D) [15]. Intermediate refractive states (−0.5 D < SE < 1.0 D) were considered emmetropic [16–18]. Astigmatic anisometropia was defined as interocular astigmatic power difference in any meridian of at least 1.0 D and was further categorized into mild (≥ 1.0 D and < 1.5 D), moderate (≥ 1.5 D and < 3.0 D) and severe (of ≥ 3.0 D) [19, 20].

### Covariates

SES was defined according to an index derived from Israel’s Central Bureau of Statistics. This is an indicator covering the subjects of demography, education, and standard of living [21]. Sixteen variables are combined into a single index, and all localities in Israel are classified into one of ten clusters: ‘1’ being the lowest and ‘10’ being the highest SES. These are further categorized into three ordinal groups (low, medium, and high). Body-mass index (BMI) classifications followed age- and gender-adjusted percentiles from the U.S Center for Disease Control and Prevention (CDC), categorized as underweight (< 5th percentile), normal (5th to 85th percentile), overweight (85th to 95th percentile) and obese (≥ 95th percentile) [22, 23].

### Statistical analysis

Continuous variables were reported as means with standard deviations (SDs), and categorical variables as proportions and percentages. For group comparisons, the Student’s t-test or ANOVA were applied to continuous variables and the Chi-Square test to categorical variables. When ANOVA yielded a significant result, Bonferroni-adjusted post hoc tests were applied. Separate logistic regression models were used to analyze the following relationships amongst hyperopic and myopic children: (a) The relationship between anisometropia degree at first examination and the presence of anisometropia at follow-up, using baseline anisometropia of 1.0–3.0 D as the reference category. (b) The relationship between the type and degree of refractive error at first examination and the presence of anisometropia at follow-up, using mild hyperopia and mild myopia as the reference category. (c) A sub-group analysis which included children with baseline astigmatic anisometropia, defined as an astigmatic power difference in any meridian of ≥ 1.0 D between both eyes. The relationship between astigmatism at first examination and the presence of anisometropia at follow-up was then analyzed, using astigmatic power of 1.0–1.5 D as the reference category. All models adjusted for socio-demographic covariates including age at baseline examination, gender, SES, BMI, and country of birth. To further evaluate potential effect modification by age, unadjusted logistic regression analyses for models (a), (b), and (c) were repeated in children stratified by age at baseline (< 4 years vs. ≥ 4 years). Results are presented as adjusted odds ratios (ORs) and their 95% confidence intervals (95% CI). P-values reported for all regression models are unadjusted for multiple comparisons. Multiple comparisons were addressed using the Benjamini–Hochberg false discovery rate (FDR) correction, and all statistically significant associations remained significant after correction. Multicollinearity was ruled out, as all covariates demonstrated a variance inflation factor (VIF) below 1.5. A p-value < 0.05 was considered statistically significant unless otherwise specified. For all ANOVA post hoc comparisons, the reported p-values are Bonferroni-adjusted. Analyses were performed using SPSS software version 29.0 (IBM SPSS Statistics).

## Results

The MHS database encompassed 35,969 children aged one to six years with a minimum two-year follow-up between 2012 and 2022. Of these, 0.3% (*n* = 115) were excluded due to chromosomal anomalies, ocular trauma, keratoconus, and cataract, with the remaining 35,854 children eligible for refractive data analysis. Of these, 6.6% (*n* = 2,358, 51.7% boys) were anisometropic at presentation and composed the group study. The mean age at the first examination was 3.7 ± 1.4 years. The mean follow-up period was 5.14 ± 2.35 years.

The baseline characteristics of the group study are presented in Table 1. At the first examination, the mean SE difference was 1.8 ± 1.3 D. The rates of hyperopia and myopia were 85.4% (*n* = 2,014) and 12.2% (*n* = 287), respectively. The mean BMI was 16.1 ± 2.0kg/m^2^. Seventy-two percent had a normal weight, 13.8% were overweight, 10.0% were obese, and 4.3% were underweight. SES distribution was as follows: medium (55.2%), high (24.9%), and low (19.9%). The majority of children were natives (98.5%).

**Table 1:** Baseline characteristics of the 2,358 anisometropic children

	Hyperopia			Emmetropia	Myopia			Total	*P* value
	Mild (≥ 1 D, < 3 D)	Moderate (≥ 3 D, < 5 D)	Severe (≥ 5 D)	(> −0.5 D, < 1 D)	Mild (> −3 D, ≤ −0.5 D)	Moderate (> −6 D, ≤ −3 D)	Severe (≤ −6 D)		
Participants, n	862	702	450	57	188	73	26	2358	
Gender, % (*n*)									0.590
Female	50.2 (433)	48.7 (342)	46.2 (208)	38.6 (22)	47.3 (89)	45.2 (33)	50.0 (13)	48.3 (1140)	
Male	49.8 (429)	51.3 (360)	53.8 (242)	61.4 (35)	52.7 (99)	54.8 (40)	50.0 (13)	51.7 (1218)	
Mean age* ± SD (years)	3.5 ± 1.5	3.7 ± 1.4	3.8 ± 1.3	3.7 ± 1.4	3.9 ± 1.4	4.0 ± 1.3	3.9 ± 1.4	3.7 ± 1.4	< 0.001
Mean SE difference* ± SD (D)	1.4 ± 0.6	1.7 ± 0.8	2.4 ± 2.1	1.1 ± 0.2	1.7 ± 0.8	2.8 ± 1.9	3.8 ± 3.1	1.8 ± 1.3	< 0.001
Mean BMI* ± SD (kg/m^2^)	16.1 ± 2.0	16.1 ± 1.9	15.9 ± 1.8	16.4 ± 3.5	16.1 ± 1.9	16.1 ± 2.2	17.3 ± 3.3	16.1 ± 2.0	0.044
BMI categories, %									0.188
Underweight	4.6	2.6	6.3	3.6	0.0	7.4	3.5	4.3	
Normal weight	72.0	73.6	71.4	67.3	70.8	69.1	70.5	72.0	
Overweight	14.4	14.7	12.1	12.7	16.7	7.4	13.9	13.8	
Obese	9.0	9.1	10.2	16.4	12.5	16.2	12.1	10.0	
Socioeconomic status, %									< 0.001
High	30.2	26.0	15.5	26.3	8.0	16.4	23.9	24.9	
Medium	54.4	54.9	56.5	56.1	56.0	53.4	57.4	55.2	
Low	15.4	19.1	28.0	17.5	36.0	30.1	18.6	19.9	
Native/immigrant, %									0.122
Native	98.5	98.7	98.0	100.0	98.9	100.0	92.3	98.5	
Immigrant	1.5	1.3	2.0	0.0	1.1	0.0	7.7	1.5	

*BMI* body mass index, *D* diopters, *SD* standard deviation, *SE* spherical equivalent

^*^See supplementary material for post hoc comparisons

**Fig. 1:**
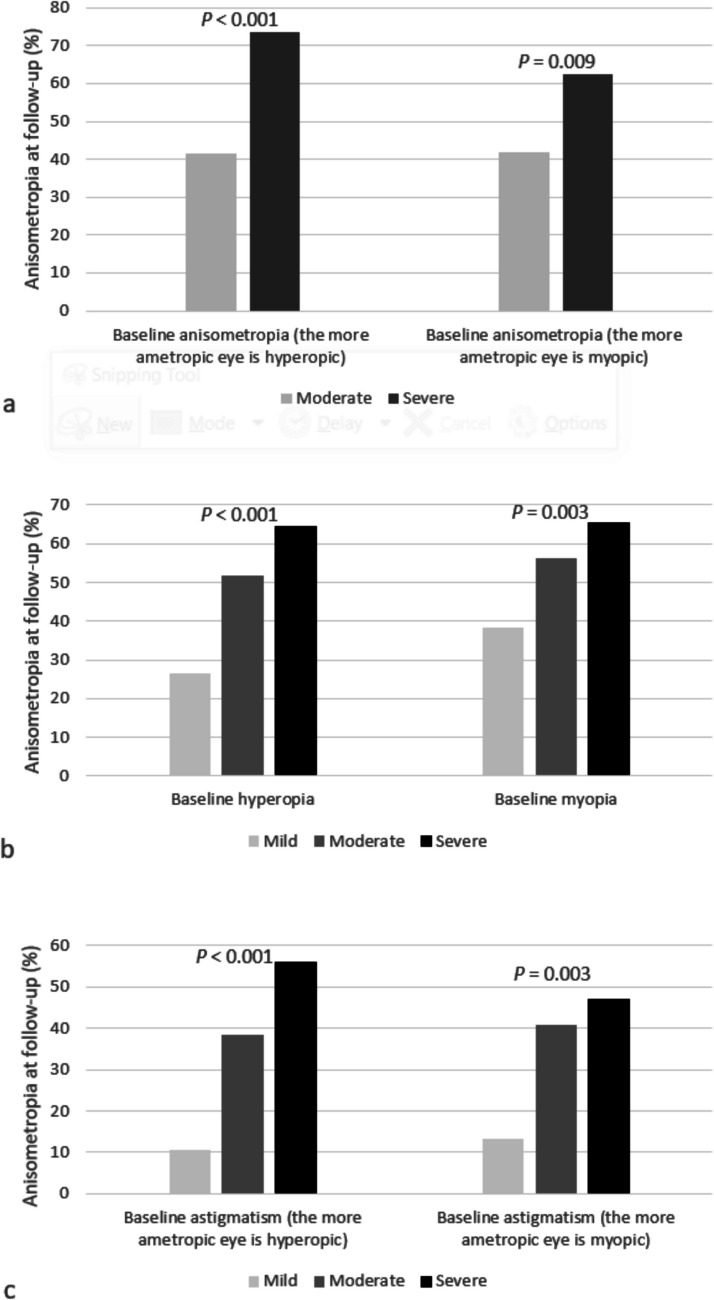
Type and degree (mild, moderate, severe) of refractive error at first examination and the prevalence of anisometropia at follow-up amongst anisometropic children aged one to six years (mean follow-up period of 5.14 ± 2.35 years)

**Fig. 2:**
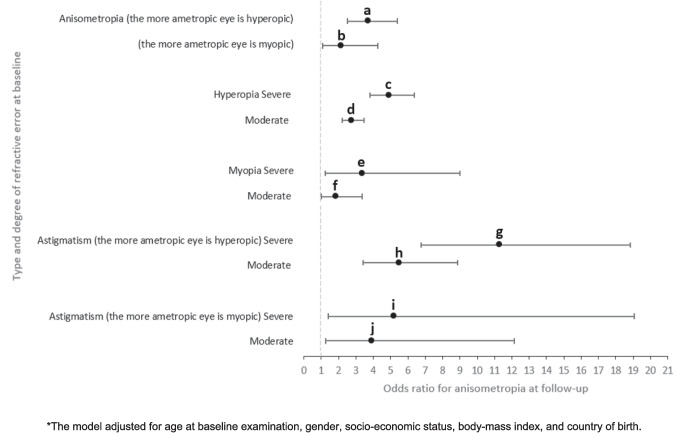
Type and degree of refractive error at first examination and the likelihood (adjusted odds ratio) of anisometropia at follow-up amongst anisometropic children aged one to six years (mean follow-up period of 5.14 ± 2.35 years)

The relationships amongst myopic and hyperopic children were as follows:

- Higher degree of anisometropia at the first examination was associated with a higher prevalence of anisometropia at the last follow-up. Those with a severe baseline anisometropia of ≥ 3.0 D had a higher prevalence of anisometropia at the last follow-up compared to those with a moderate baseline anisometropia between ≥ 1.0 D and < 3.0 D, with the more ametropic eye being hyperopic (73.5% vs. 41.4%, respectively, *p* < 0.001, Table 2, Fig. 1a) or myopic (62.5% vs. 41.8%, respectively, *p* = 0.009, Table 3, Fig. 1a). In the regression models, adjusted for age at baseline examination, gender, SES, BMI, and country of birth, OR for anisometropia at last follow-up increased with greater levels of baseline anisometropia, with the more ametropic eye being hyperopic (OR = 3.75, 95% CI 2.57–5.48, Table 2, Fig. 2; bar a) or myopic (OR = 2.19, 95% CI 1.11–4.33, Table 3, Fig. 2; bar b).

**Table 2:** The relationship between anisometropia degree (with the more ametropic eye being hyperopic) at first examination and the presence of anisometropia at follow-up

	Total	Anisometropia degree		*P* value
		Moderate (≥ 1 D, < 3 D)	Severe (≥ 3 D)	
Examinees, *n*	2014	1859	155	
Mean age at follow-up ± SD (years)	8.8 ± 2.8	8.7 ± 2.8	9.0 ± 2.4	0.227
Mean follow-up ± SD (years)	5.1 ± 2.3	5.1 ± 2.3	5.1 ± 2.1	0.691
Mean interocular SE difference^†^ at follow-up ± SD (Diopters)	1.1 ± 1.3	1.0 ± 1.1	2.6 ± 2.1	< 0.001
With anisometropia at follow-up, % (*n*)	43.9 (884)	41.4 (770)	73.5 (114)	< 0.001
Unadjusted OR for anisometropia at follow-up		reference	3.93	
95% CI			2.72–5.69	
*P* value			< 0.001	
Adjusted* OR for anisometropia at follow-up		reference	3.75	
95% CI			2.57–5.48	
*P* value			< 0.001	

*CI* confidence interval, *OR* odds ratio, *SD* standard deviation, *SE* spherical equivalent

^*^The model was adjusted for age at baseline examination, gender, socioeconomic status, body-mass index, and country of birth

^†^*P* values for continuous variables were calculated using independent samples t-tests

**Table 3:** The relationship between anisometropia degree (with the more ametropic eye being myopic) at first examination and the presence of anisometropia at follow-up

	Total	Anisometropia degree		*P* value
		Moderate (≥ 1 D, < 3 D)	Severe (≥ 3 D)	
Examinees, n	287	239	48	
Mean age at follow-up ± SD (years)	9.1 ± 2.9	9.0 ± 2.8	9.7 ± 2.7	0.124
Mean follow-up ± SD (years)	5.2 ± 2.5	5.1 ± 2.5	5.7 ± 2.5	0.121
Mean interocular SE difference^†^ at follow-up ± SD (Diopters)	1.4 ± 1.8	1.1 ± 1.2	2.9 ± 2.9	< 0.001
With anisometropia at follow-up, % (*n*)	45.3 (130)	41.8 (100)	62.5 (30)	0.009
Unadjusted OR for anisometropia at follow-up		reference	2.32	
95% CI			1.22–4.39	
*P* value			0.01	
Adjusted* OR for anisometropia at follow-up		reference	2.19	
95% CI			1.11–4.33	
*P* value			0.02	

*CI* confidence interval, *OR* odds ratio, *SD* standard deviation, *SE* spherical equivalent

^*^The model was adjusted for age at baseline examination, gender, socioeconomic status, body-mass index, and country of birth

^†^*P* values for continuous variables were calculated using independent samples t-tests
- Higher degree of ametropia at the first examination was associated with a higher prevalence of anisometropia at the last follow-up. Those with a severe baseline hyperopia of ≥ 5.0 D had a higher prevalence of anisometropia at the last follow-up compared to those with a moderate baseline hyperopia between < 5.0 D and ≥ 3.0 D and compared to those with a mild baseline hyperopia between < 3.0 D and ≥ 1.0 D, (64.7% vs. 51.9% vs. 26.6%, respectively, p < 0.001, eTable 4, Fig. 1b). Adjusted OR for anisometropia at last follow-up increased with a severe baseline hyperopia compared to moderate (OR = 4.99, 95% CI 3.85–6.45 vs. OR = 2.83, 95% CI 2.27–3.53, eTable 4, Fig. 2; bar c, d). Those with a severe baseline myopia of ≤ −6.0 D had a higher prevalence of anisometropia at the last follow-up compared to those with a moderate baseline myopia between > −6.0 D and ≤ −3.0 D and compared to those with a mild baseline myopia between > −3.0 D and ≤ −0.5 D (65.4% vs. 56.2% vs. 38.3%, respectively, *p* = 0.003, eTable 5, Fig. 1b). Adjusted OR for anisometropia at last follow-up increased with a severe baseline myopia compared to moderate (OR = 3.43, 95% CI 1.29–9.11 vs. OR = 1.90, 95% CI 1.06–3.40, eTable 5, Fig. 2; bar e, f).
- In the sub-group analysis of children with baseline astigmatic anisometropia, higher degree of astigmatism at the first examination was associated with a higher prevalence of anisometropia at the last follow-up. Those with a severe baseline astigmatism of ≥ 3.0 D had a higher prevalence of anisometropia at the last follow-up compared to those with a moderate baseline astigmatism between < 3.0 D and ≥ 1.5 D and compared to those with a mild baseline astigmatism between < 1.5 D and ≥ 1.0 D, with the more ametropic eye being hyperopic (56.1% vs. 38.4% vs. 10.7%, respectively, p < 0.001, eTable 6, Fig. 1c) or myopic (47.2% vs. 40.8% vs. 13.2%, respectively, *p* = 0.003, eTable 7, Fig. 1c). Adjusted OR for anisometropia at the last follow-up increased with severe baseline astigmatism compared to moderate, with the more ametropic eye being hyperopic (OR = 11.45, 95% CI 6.86–19.12 vs. OR = 5.60, 95% CI 3.48–9.00, eTable 6, Fig. 2; bar g, h) or myopic (OR = 5.30, 95% CI 1.45–19.33 vs. OR = 4.00, 95% CI 1.30–12.31, eTable 7, Fig. 2; bar i, j).

### Age-stratified analysis

Unadjusted logistic regression analyses were repeated after stratifying children by age at baseline (< 4 years vs. ≥ 4 years) to examine whether the association between baseline anisometropia severity and anisometropia at follow-up differed by developmental stage. In hyperopic children, severe anisometropia (≥ 3.0 D) was significantly associated with higher odds of anisometropia at follow-up in both the 1–3 year group (*n* = 1,152; OR = 4.11, 95% CI 2.39–7.08, *p* < 0.001) and the ≥ 4 year group (*n* = 862; OR = 3.55, 95% CI 2.15–5.88, *p* < 0.001), using moderate anisometropia (≥ 1.0 D to < 3.0 D) as the reference. Among myopic children, a similar trend was observed, although associations did not reach statistical significance—1–3 years (*n* = 143; OR = 2.39, 95% CI 0.98–5.83, *p* = 0.056) and ≥ 4 years (*n* = 144; OR = 2.29, 95% CI 0.91–5.74, *p* = 0.079). Similar findings were observed in age-stratified analyses for models (b) and (c) among hyperopic children, but not among myopic children.

## Discussion

In this cohort of 2,358 anisometropic preschool-aged children, higher degrees of baseline anisometropia, hyperopia, myopia and astigmatism, were associated with a higher prevalence of anisometropia at the last follow-up. This stands in agreement with a previous study assessing a group of adult subjects, which found an increase in the prevalence of anisometropia with increasing myopia, hyperopia and astigmatism. [24] In the regression models, greater degree of baseline anisometropia, hyperopia, myopia and astigmatism, resulted in a higher likelihood for anisometropia at the last follow-up, whether the more ametropic eye was hyperopic or myopic. This stands in partial disparity with the results of a previous small study, conducted on 30 amblyopic children with high anisometropia. In that study, overtime, a significant reduction in anisometropia was observed amongst children with myopia, whereas anisometropia amongst children with hyperopia remained constant. The authors attributed this to the dynamic refractive changes observed in children with amblyopia. Contrary to increased anisometropia in non-amblyopic children as myopia progresses, the sound (non-amblyopic) eyes of the myopic children exhibited more pronounced myopic shifts than the amblyopic eyes. However, a synchronous decrease in hyperopia was noted in both eyes of hyperopic children [25]. Another study, which investigated the progression of baseline high anisometropia (interocular SE difference of at least 4.0 D between both eyes) in myopic children, showed that anisometropic myopia progressed rapidly in the first few years of life [11]. Our study again emphasizes the importance of preschool refraction screening, in order to determine who are the children at risk of continuing anisometropia based on baseline refractive measurements. Of note, prescribing refractive correction by itself is an inherent intervention in this study (which is also subjected to variable compliance) that may impact the amount of persistent anisometropia at the follow-up visit which would have otherwise might have been even higher [26].

Infantile refractive errors can result from a faulted emmetropization process or by an initial refractive error too great to be corrected by emmetropization [27]. Such large congenital refractive errors are uncommon and often associated with genetic disorders [28, 29]. In hyperopia, refractive distribution at the age of six years is characterized by a positive skew which indicates that most hyperopia is attributed to the persistence of infantile hyperopia secondary to emmetropization failure [27]. In regard to myopia, previous work done on infant monkeys, demonstrated that peripheral hyperopic defocus induced progression of myopia and had a substantial impact on normal refractive development [30, 31]. Although a significant decline in the visual input to one eye due to eye structural pathologies, such as infantile cataract, can induce axial elongation and anisometropia, in most cases of anisometropia, there are no ophthalmic structural anomalies. A deviation from the normal emmetropization process, in the form of greater levels of baseline anisometropia, hyperopia, myopia and astigmatism, may increase the chance of anisometropia persistence, although the mechanism for this is not entirely certain.

Our study adds information regarding the astigmatic effect on anisometropia. Previous studies did not indicate whether higher prevalence of anisometropia amongst astigmats was due purely to an increase in astigmatic anisometropia or to an increase in both SE and astigmatic anisometropia [32]. In the present study, the likelihood for anisometropia at the last follow-up has increased considerably with astigmatic myopia and with astigmatic hyperopia. In a study conducted on albino patients, against-the-rule astigmatism, which is unaffected by motion defocus of horizontal nystagmus, displayed greater degree of emmetropization compared with with-the-rule astigmatism [33]. This possibly underscores the combined effect of astigmatism and hyperopia on anisometropia persistence. Earlier development of unilateral defocus in the presence of astigmatism, and astigmatic hyperopia in particular, may disrupt emmetropization to a greater extent than other types of refractive errors.

The risk of amblyopia approximately doubles in cases of anisohyperopia compared with anisomyopia of similar refractive imbalance and even minimal disparity in interocular refractive error can have profound ramifications on visual function. Mild to moderate anisometropic myopes use the more myopic eye for near targets and the less myopic eye for distant targets. Although it may result in a blurred image for distant targets, this allows for some degree of visual stimulation, reducing the risk of amblyopia. In contrast, anisometropic hyperopes can accommodate, obtaining a clear image in the less hyperopic eye, while in the more hyperopic eye, if accommodation does not compensate, vision is impaired at both near and far. This blurred image provides reduced stimulation to the visual cortex, increasing the likelihood of amblyopia development [34].

The underlying mechanisms of amblyopia involve abnormal visual experience during the critical period of visual development, which can result from foveal suppression, impaired binocular coordination, aniso-accommodative stress, lack of compensatory treatment during the critical period and more [35, 36]. These may lead to cortical deficits in which reduced neuronal activity secondary to decreased input from the eye, altered patterns of coordinated neuronal interactions, and impaired interocular excitatory and inhibitory balance, affect the primary visual cortex and higher-order visual areas [37].

Yet, the minimal amount of anisometropia that calls for refractive correction remains unclear. As anisometropia is a common cause of amblyopia [38], early detection and intervention are crucial for managing anisometropia's developmental impact. Many pediatric health and opthalmological organizations around the world have conducted pediatric vision screening programs [39]. While variations exist in terms of timing, intervals of exams and the staff responsible for their execution, it is agreed that vision screening for amblyopia and its associated risk factors should begin in early childhood, typically between the ages of three and five years [40, 41].

Our findings could aid clinicians in targeting patients who are at increased risk of anisometropia persistence and place them on a more intensive follow-up protocol, early refractive correction, or alternative prophylactic therapy. The exact nature of such intervention and long-term management should be sought in future designated studies. However, with optical treatments utilizing peripheral defocus to mitigate myopia progression now available [42], it may be possible to employ analogous optical solutions to address anisometropia as well. This could theoretically be achieved by applying peripheral myopic defocus to the more myopic eye and peripheral hyperopic defocus to the more hyperopic eye, through the use of glasses or contact lenses.

The strengths of this study include a large sample size (*n* = 2,358) of anisometropic young children. Second, the group study covered a broad range of refractive errors including astigmatic anisometropia. Third, the cohort was comprised of a relatively uniformed population which served in avoiding potential demographic discrepancies. Our study does have several limitations. First, the context of the first refractive examination was not accounted for, as whether it was part of a routine screening examination or following a medical referral regarding a specific ophthalmic pathology which may cause bias. Second, data were obtained from an electronic database containing refractive examinations done by different personnel. Third, neighborhood-level SES measures are prone to ecological fallacy. Fourth, while the common practice is to use cyclopentolate 1%, it is possible that some ophthalmologists used tropicamide for cycloplegic refraction, which rises the possibility of residual accommodation. In addition, residual accommodation and refractive error measurements in general could vary depending on the measurement technique. Nevertheless, our attention on exploring relative changes in refraction between both eyes, rather than only evaluating the absolute values of refraction in each eye, mitigates, at least partially, this variation. Finaly, while emmetropization is largely complete by the age of one year, the process can extend, albeit more slowly, until three years of age with minor adjustments continuing until 18 years of age [43]. This may weaken the relationships found in this study as they are drawn from a period where refractive error measurements may be less stable. To overcome this limitation, we adjusted the multivariate analyses to age at baseline examination. We also conducted an age-stratified analysis which demonstrated that in hyperopic children, severe anisometropia was significantly associated with higher odds of anisometropia at follow-up. Among myopic children, a similar trend was observed, although associations did not reach statistical significance, possibly due to limited sample size. Similarly, it is feasible that our cohort was too young to demonstrate relationships with myopia to the fullest, which can become evident only at a later age.

In conclusion, higher degrees of baseline anisometropia and ametropia in preschool-aged children were associated with the prevalence of future anisometropia. The likelihood for anisometropia at the last follow-up has increased with greater levels of baseline anisometropia, hyperopia, myopia and astigmatism. Further research will focus on how to practically implement these findings in clinical practice, in order to target patients who are at increased risk of anisometropia persistence and place them on a closer follow-up or earlier treatment.

## Supplementary Information

Below is the link to the electronic supplementary material.

Supplementary file1 (DOCX 22.0 KB)
