# Supplementary material for: Refractive factors affecting the persistence of anisometropia in preschool-aged children
Source: Graefes Arch Clin Exp Ophthalmol. 2025 Jun 27;263(10):2935–44. doi: 10.1007/s00417-025-06891-w (PMC12583331; doi:10.1007/s00417-025-06891-w)
Supplement: Supplementary file 1 — Supplementary file1 (DOCX 22.0 KB) [file 417_2025_6891_MOESM1_ESM.docx]

Supplementary material for Table 1

Post hoc comparisons:

A statistically significant age differences were found between Mild Myopia and Mild Hyperopia (p = 0.020) and between Mild Myopia and Moderate Hyperopia (p = 0.023) (Bonferroni-adjusted).

A statistically significant differences in mean SE difference were found between Severe Hyperopia and each of the following groups: Mild Myopia (p = 0.035), Moderate Myopia (p < 0.001), Severe Myopia (p < 0.001), Mild Hyperopia (p < 0.001), Moderate Hyperopia (p < 0.001), and Emmetropia (p < 0.001). Additional significant differences were observed between Moderate Myopia and Mild Myopia (p < 0.001), Emmetropia (p = 0.009), Mild Hyperopia (p < 0.001), and Moderate Hyperopia (p < 0.001) (Bonferroni-adjusted).

A statistically significant difference in BMI was found between Severe Hyperopia and Severe Myopia (p = 0.025; Bonferroni-adjusted).

| **eTable 4. The relationship between the degree of hyperopia at first examination and the presence of anisometropia at follow-up.** | | | | | |
| --- | --- | --- | --- | --- | --- |
|  | **Total** | **Hyperopia degree** | | | **P value** |
|  |  | **Mild (≥ 1 D, < 3 D)** | **Moderate (≥ 3 D, < 5 D)** | **Severe (≥ 5 D)** |  |
| Examinees, n | 2014 | 862 | 702 | 450 |  |
| Mean age at follow-up ± SD (years) | 8.8 ± 2.8 | 8.7 ± 2.9 | 8.7 ± 2.8 | 9.0 ± 2.6 | 0.148 |
| Mean follow-up ± SD (years) | 5.1 ± 2.3 | 5.2 ± 2.3 | 5.0 ± 2.3 | 5.2 ± 2.3 | 0.098 |
| Mean interocular SE difference^†^ at follow-up ± SD (Diopters) | 1.1 ± 1.3 | 0.8 ± 1.1 | 1.3 ± 1.2 | 1.7 ± 1.5 | <0.001 |
| With anisometropia at follow-up, % (n) | 43.9 (884) | 26.6 (229) | 51.9 (364) | 64.7 (291) | <0.001 |
| Unadjusted OR for anisometropia at follow-up |  | reference | 2.98 | 5.06 |  |
| 95% CI |  |  | 2.41-3.68 | 3.96-6.47 |  |
| P value |  |  | <0.001 | <0.001 |  |
| Adjusted* OR for anisometropia at follow-up |  | reference | 2.83 | 4.99 |  |
| 95% CI |  |  | 2.27-3.53 | 3.85-6.45 |  |
| P value |  |  | <0.001 | <0.001 |  |
| CI, confidence interval; OR, odds ratio; SD, standard deviation; SE, spherical equivalent. | | | | | |
| *The model was adjusted for age at baseline examination, gender, socioeconomic status, body-mass index, and country of birth.  †P values for continuous variables were calculated using one-way ANOVA with Bonferroni-adjusted post hoc comparisons; all pairwise comparisons were statistically significant at p < 0.05. | | | | | |

| **eTable 5. The relationship between the degree of myopia at first examination and the presence of anisometropia at follow-up.** | | | | | |
| --- | --- | --- | --- | --- | --- |
|  | **Total** | **Myopia degree** | | | **P value** |
|  |  | **Mild (> -3 D, ≤ -0.5 D)** | **Moderate (> -6 D, ≤ -3 D)** | **Severe (≤ -6 D)** |  |
| Examinees, n | 287 | 188 | 73 | 26 |  |
| Mean age at follow-up ± SD (years) | 9.1 ± 2.9 | 8.8 ± 2.8 | 9.4 ± 2.9 | 9.8 ± 3.2 | 0.128 |
| Mean follow-up ± SD (years) | 5.2 ± 2.5 | 5.0 ± 2.5 | 5.4 ± 2.5 | 6.0 ± 2.7 | 0.105 |
| Mean interocular SE difference^†^ at follow-up ± SD (Diopters) | 1.4 ± 1.8 | 1.0 ± 1.2 | 1.8 ± 1.8 | 2.9 ± 3.4 | <0.001 |
| With anisometropia at follow-up, % (n) | 45.3 (130) | 38.3 (72) | 56.2 (41) | 65.4 (17) | 0.003 |
| Unadjusted OR for anisometropia at follow-up |  | reference | 2.06 | 3.04 |  |
| 95% CI |  |  | 1.19-3.57 | 1.29-7.19 |  |
| P value |  |  | 0.010 | 0.011 |  |
| Adjusted* OR for anisometropia at follow-up |  | reference | 1.90 | 3.43 |  |
| 95% CI |  |  | 1.06-3.40 | 1.29-9.11 |  |
| P value |  |  | 0.031 | 0.013 |  |
| CI, confidence interval; OR, odds ratio; SD, standard deviation; SE, spherical equivalent. | | | | | |
| *The model was adjusted for age at baseline examination, gender, socioeconomic status, body-mass index, and country of birth.  †P values for continuous variables were calculated using one-way ANOVA with Bonferroni-adjusted post hoc comparisons; all pairwise comparisons were statistically significant at p < 0.05. | | | | | |

| **eTable 6. The relationship between the degree of astigmatism (with the more ametropic eye being hyperopic) at first examination and the presence of anisometropia at follow-up.** | | | | | |
| --- | --- | --- | --- | --- | --- |
|  | **Total** | **Astigmatism degree** | | | **P value** |
|  |  | **Mild (≥ 1 D, < 1.5 D)** | **Moderate (≥ 1.5 D, < 3 D)** | **Severe (≥ 3 D)** |  |
| Examinees, n | 1050 | 252 | 552 | 246 |  |
| Mean age at follow-up ± SD (years) | 8.8 ± 2.8 | 8.5 ± 2.9 | 8.8 ± 2.9 | 9.3 ± 2.6 | 0.969 |
| Mean follow-up ± SD (years) | 5.2 ± 2.4 | 5.2 ± 2.4 | 5.2 ± 2.4 | 5.3 ± 2.2 | 0.369 |
| Mean interocular astigmatic difference^†^ at follow-up ± SD (Diopters) | 0.9 ± 1.4 | 0.3 ± 0.4 | 0.9 ± 1.7 | 1.3 ± 1.1 | <0.001 |
| With anisometropia at follow-up, % (n) | 35.9 (377) | 10.7 (27) | 38.4 (212) | 56.1 (138) | <0.001 |
| Unadjusted OR for cylindrical anisometropia at follow-up |  | reference | 5.20 | 10.65 |  |
| 95% CI |  |  | 3.37-8.02 | 6.64-17.07 |  |
| P value |  |  | <0.001 | <0.001 |  |
| Adjusted* OR for cylindrical anisometropia at follow-up |  | reference | 5.60 | 11.45 |  |
| 95% CI |  |  | 3.48-9.00 | 6.86-19.12 |  |
| P value |  |  | <0.001 | <0.001 |  |
| CI, confidence interval; OR, odds ratio; SD, standard deviation. | | | | | |
| *The model was adjusted for age at baseline examination, gender, socioeconomic status, body-mass index, and country of birth.  †P values for continuous variables were calculated using one-way ANOVA with Bonferroni-adjusted post hoc comparisons; all pairwise comparisons were statistically significant at p < 0.05. | | | | | |

| **eTable 7. The relationship between the degree of astigmatism (with the more ametropic eye being myopic) at first examination and the presence of anisometropia at follow-up.** | | | | | |
| --- | --- | --- | --- | --- | --- |
|  | **Total** | **Astigmatism degree** | | | **P value** |
|  |  | **Mild (≥ 1 D, < 1.5 D)** | **Moderate (≥ 1.5 D, < 3 D)** | **Severe (≥ 3 D)** |  |
| Examinees, n | 145 | 38 | 71 | 36 |  |
| Mean age at follow-up ± SD (years) | 9.0 ± 2.8 | 9.4 ± 2.5 | 9.0 ± 2.8 | 8.5 ± 3.3 | 0.733 |
| Mean follow-up ± SD (years) | 5.1 ± 2.5 | 5.3 ± 2.4 | 5.0 ± 2.5 | 5.2 ± 2.7 | 0.599 |
| Mean interocular astigmatic difference at follow-up ± SD (Diopters) | 0.8 ± 0.9 | 0.5 ± 0.4 | 0.9 ± 0.8 | 1.1 ± 1.2 | 0.754 |
| With anisometropia at follow-up, % (n) | 35.2 (51) | 13.2 (5) | 40.8 (29) | 47.2 (17) | 0.003 |
| Unadjusted OR for cylindrical anisometropia at follow-up |  | reference | 4.56 | 5.91 |  |
| 95% CI |  |  | 1.59-13.06 | 1.88-18.57 |  |
| P value |  |  | 0.005 | 0.002 |  |
| Adjusted* OR for cylindrical anisometropia at follow-up |  | reference | 4.00 | 5.30 |  |
| 95% CI |  |  | 1.30-12.31 | 1.45-19.33 |  |
| P value |  |  | 0.015 | 0.012 |  |
| CI, confidence interval; OR, odds ratio; SD, standard deviation. | | | | | |
| *The model was adjusted for age at baseline examination, gender, socioeconomic status, body-mass index, and country of birth. | | | | | |
